# Major alterations in the mononuclear phagocyte landscape associated with COVID-19 severity

**DOI:** 10.1073/pnas.2018587118

**Published:** 2021-01-21

**Authors:** Egle Kvedaraite, Laura Hertwig, Indranil Sinha, Andrea Ponzetta, Ida Hed Myrberg, Magda Lourda, Majda Dzidic, Mira Akber, Jonas Klingström, Elin Folkesson, Jagadeeswara Rao Muvva, Puran Chen, Sara Gredmark-Russ, Susanna Brighenti, Anna Norrby-Teglund, Lars I. Eriksson, Olav Rooyackers, Soo Aleman, Kristoffer Strålin, Hans-Gustaf Ljunggren, Florent Ginhoux, Niklas K. Björkström, Jan-Inge Henter, Mattias Svensson, Karolinska KI/K COVID-19 Study Group

**Affiliations:** ^a^Center for Infectious Medicine, Department of Medicine Huddinge, Karolinska Institutet, Karolinska University Hospital, 171 77 Stockholm, Sweden;; ^b^Childhood Cancer Research Unit, Department of Women’s and Children’s Health, Karolinska Institutet, 171 77 Stockholm, Sweden;; ^c^Department of Infectious Diseases, Karolinska University Hospital, 171 77 Stockholm, Sweden;; ^d^Department of Physiology and Pharmacology, Section for Anesthesiology and Intensive Care, Karolinska Institutet, 171 77 Stockholm, Sweden;; ^e^Function Perioperative Medicine and Intensive Care, Karolinska University Hospital, 171 77 Stockholm, Sweden;; ^f^Division of Anesthesiology and Intensive Care, Department of Clinical Science, Intervention, and Technology, Karolinska Institutet, 141 52 Huddinge, Sweden;; ^g^Division of Infectious Diseases, Department of Medicine Huddinge, Karolinska Institutet, 171 77 Stockholm, Sweden;; ^h^Singapore Immunology Network, Agency for Science, Technology and Research, BIOPOLIS, 138648 Singapore, Singapore;; ^i^Shanghai Institute of Immunology, Shanghai JiaoTong University School of Medicine, 200240 Shanghai, China;; ^j^Translational Immunology Institute, SingHealth Duke-National University of Singapore Academic Medical Centre, 168753 Singapore, Singapore;; ^k^Pediatric Oncology, Theme of Children’s Health, Karolinska University Hospital, 171 77 Stockholm, Sweden

**Keywords:** pre-DCs, DCs, monocytes, COVID-19

## Abstract

Dendritic cells (DCs) and monocytes are crucial mediators of innate and adaptive immune responses during viral infection, but misdirected responses by these cells may contribute to immunopathology. Here, we performed high-dimensional flow cytometry-analysis focusing on mononuclear phagocyte (MNP) lineages in SARS-CoV-2–infected patients with moderate and severe COVID-19. We provide a deep and comprehensive map of the MNP landscape in COVID-19. A redistribution of monocyte subsets toward intermediate monocytes and a general decrease in circulating DCs was observed in response to infection. Severe disease coincided with the appearance of monocytic myeloid-derived suppressor cell-like cells and a higher frequency of pre-DC2. Furthermore, phenotypic alterations in MNPs, and their late precursors, were cell-lineage–specific and associated either with the general response against SARS-CoV-2 or COVID-19 severity. This included an interferon-imprint in DC1s observed in all patients and a decreased expression of the coinhibitory molecule CD200R in pre-DCs, DC2s, and DC3 subsets of severely sick patients. Finally, unsupervised analysis revealed that the MNP profile, alone, pointed to a cluster of COVID-19 nonsurvivors. This study provides a reference for the MNP response to SARS-CoV-2 infection and unravels mononuclear phagocyte dysregulations associated with severe COVID-19.

## Significance

While broad efforts toward getting an overview of immune cell and soluble factor alterations in COVID-19 are under way, a deep and comprehensive understanding of the mononuclear phagocyte system, including circulating progenitors, is still largely lacking. This study provides a reference for the mononuclear phagocyte response to SARS-CoV-2 infection and unravels mononuclear phagocyte dysregulations associated with severe COVID-19.

The current COVID-19 pandemic has claimed more than one million lives worldwide during the first 10 mo of 2020 (https://www.who.int/emergencies/diseases/novel-coronavirus-2019). The clinical presentation of COVID-19 can vary from asymptomatic to life-threatening acute respiratory distress syndrome and multiple organ failure. Additional potentially lethal complications include profound coagulation abnormalities associated with systemic thrombogenicity combined with a hyperinflammatory state (1, 2). Despite a steadily growing body of information regarding the host immune response to SARS-CoV-2 infection and the pathophysiology behind COVID-19, it is still unclear why certain patients enter the detrimental courses of the disease while others merely present with mild or no symptoms (2). Thus, there is an urgent need to characterize, in-depth, the immunological and inflammatory aspects of SARS-CoV-2 infection and ensuing COVID-19.

Mononuclear phagocytes (MNPs) in peripheral blood comprise dendritic cells (DCs) and monocytes, both with central roles in orchestrating the induction of innate and adaptive immune responses. MNPs are at the front line during an infection, capable of recognizing, processing, and presenting antigens to immune cells and at the same time produce cytokines and regulate immune responses (3, 4). They are classified with respect to their ontogeny and specialized functions. Monocytes are rapidly recruited to the site of infection, and can be split into three major populations: that is, classical, intermediate, and nonclassical monocytes (5–7). Monocytes provide both proinflammatory and resolving functions in conjunction with other immune cells present at the site of infection (8, 9). DCs are also a heterogeneous group of cells divided into conventional DCs (cDCs) and type I interferon- (IFN-) producing plasmacytoid DCs (pDCs) (10, 11). Among cDCs, cDC1s are specialized in cross-presenting antigens to CD8^+^ T cells while cDC2s initiate T-helper cell responses, both of which are essential for successful viral clearance (12). cDC1s constitute a discrete population of cells identified based on expression of highly specific surface proteins, such as CLEC9A. cDC2s are more heterogeneous as evident by the functionally distinct CD5^+^ DC2 and CD5^−^ DC3 subsets, among which inflammatory DC3s positive for the classically monocyte restricted marker CD14 are found (13–16). In addition, circulating DC precursors, described as pre-DC (17) or AS DC (AXL^+^SIGLEC6^+^ DCs) (18), have recently been discovered.

Although MNPs play a central role in the defense against infections, misdirected MNP responses may also contribute to immunopathology. Indeed, the importance of monocytes, monocyte-derived cells, and DCs in COVID-19 pathogenesis is emerging (19–27). Yet, a detailed understanding of developmental and phenotypic alterations, especially with respect to DC lineages and their circulating precursors in COVID-19, is lacking. Moreover, clues on how the altered MNP compartment in COVID-19 is linked to host responses against the virus, disease severity, clinical complications, and ultimate disease outcome are still limited. Here, we performed a deep profiling of the peripheral blood MNP landscape using 25-color flow cytometry, integrated with publicly available single-cell transcriptional data from lung tissue, as well as soluble inflammatory serum factors in SARS-CoV-2–infected patients with moderate and severe COVID-19. Together, these results provide a comprehensive resource of the MNP landscape in response to SARS-CoV-2 infection and ensuing COVID-19.

## Results

### SARS-CoV-2 Infection Causes Declining Numbers of Circulating cDCs, Their Progenitors, and pDCs.

**Fig. 1.**
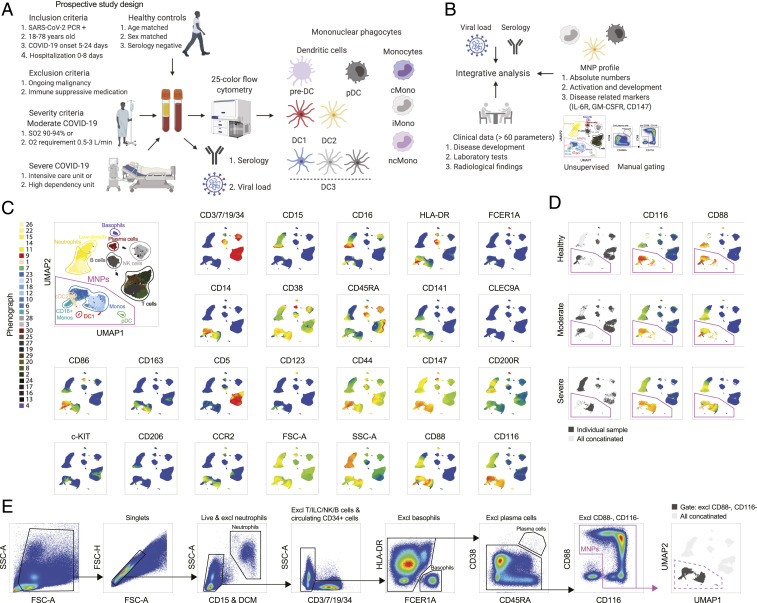
Clinical and experimental study design and analytical pipeline. (*A*) Experimental design. (*B*) Analytical pipeline. (*C*) Flow cytometry data in a UMAP plot with color-coded Phenograph clusters with cell identities established based on the displayed markers; 500,000 live cells from one representative individual from each group (healthy, moderate, severe) are shown after concatenation. (*D*) Representative individual from each cohort presented in a UMAP; CD116 and CD88 expression highlighted. (*E*) Gating strategy to identify MNPs, after exclusion steps detected in the gate defined as CD88^+^ and/or CD116^+^ (i.e., excluding CD88^−^, CD116^−^ cells), and finally projected back to the UMAP.

To study the immune profile of MNPs in SARS-CoV-2 infection, hospitalized patients with ongoing moderate or severe COVID-19 were recruited based on inclusion and exclusion criteria early on in the course of their disease (Fig. 1*A* and *SI Appendix*, *Material and Methods* and Tables S1 and S2). There was no difference in time from onset of symptoms until hospital admission, nor until study sampling, between the two severity groups (*SI Appendix*, Table S2). An integrative analysis approach was taken where the detailed phenotype of distinct MNP lineages was analyzed in relation to the clinical disease status, combining supervised and unsupervised analysis strategies, with the aim to comprehensively chart the response of circulating MNPs in response to SARS-CoV-2 infection and severity of COVID-19 (Fig. 1*B*). As immune homeostasis is significantly disrupted in COVID-19, canonical lineage markers, such as HLA-DR, normally used to identify discrete subsets of MNPs, are altered in expression (19). As a point of departure, we designed an MNP-focused 25-color flow cytometry panel (Fig. 1*C*). After exclusion of granulocytes (CD15^+^), NK cells (CD7^+^), ILCs (CD7^+^), B cells (CD19^+^), T cells (CD3^+^), circulating early progenitors (CD34^+^), basophils (FCER1A^+^HLA-DR^−^), and plasma cells (CD38^+^CD45RA^+^CD19^low^), the total MNPs were identified among the cells defined as CD88^+^ and/or CD116^+^ (Fig. 1 *D* and *E*).

**Fig. 2.**
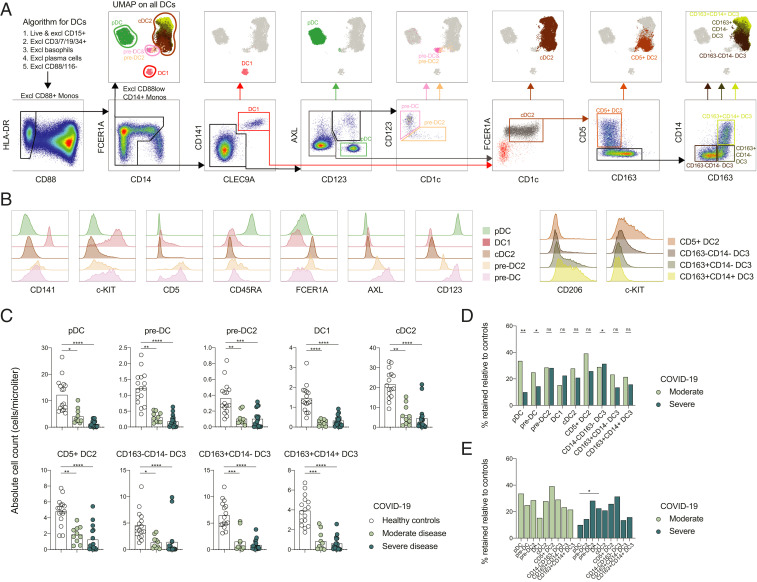
Circulating cDCs, their progenitors, and pDCs decline in numbers in COVID-19 irrespective of disease severity. (*A*) DC gating strategy (*Lower*) projected to the UMAP (*Upper*) in all concatenated samples. (*B*) Key marker expression in the DCs presented across the identified DC subsets. (*C*) Absolute DC numbers in healthy controls, and moderate and severe COVID-19 patients. (*D*) Absolute DC numbers, each patient related to the mean of the controls as percentage of maintained cells and compared in moderate vs. severe patients for each DC population. (*E*) Percentage of maintained cells, calculated as described in *D*, compared among DC subsets in moderate and severe patients separately. Statistical evaluation using a Kruskal–Wallis test and Dunn’s multiple comparisons test (*C*), Mann–Whitney *U* test (*D*), and Friedman test (*E*). *P* values: *P < 0.05, **P < 0.01, ***P < 0.001, ****P < 0.0001; ns, not significant.

The MNP identification strategy allowed clear visualization of DC1, pre-DC and pre-DC2, cDC2, CD5^+^ DC2, and three subsets of DC3 cells (CD163^−^CD14^−^, CD163^−^CD14^+^, and CD163^+^CD14^+^) (Fig. 2*A*). This approach was further validated by assessing DC subset-specific markers (Fig. 2*B*). Analysis of absolute numbers of the identified DC subsets revealed that all subsets were decreased in SARS-CoV-2–infected patients compared to healthy controls (Fig. 2*C*). Only minor differences were observed between the different disease severity groups with respect to the reduction in pDCs and pre-DC. If anything, it was more profound in the severe COVID-19 patients (Fig. 2*D*), and no major differences between the different DC sublineages were detected (Fig. 2*E*). These results indicate that the number of circulating cDCs, their late progenitors, and pDCs are reduced during SARS-CoV-2 infection.

### DC Response to SARS-CoV-2 Infection and Affected Developmental-Related Phenotypes Detected in Severe COVID-19.

**Fig. 3.**
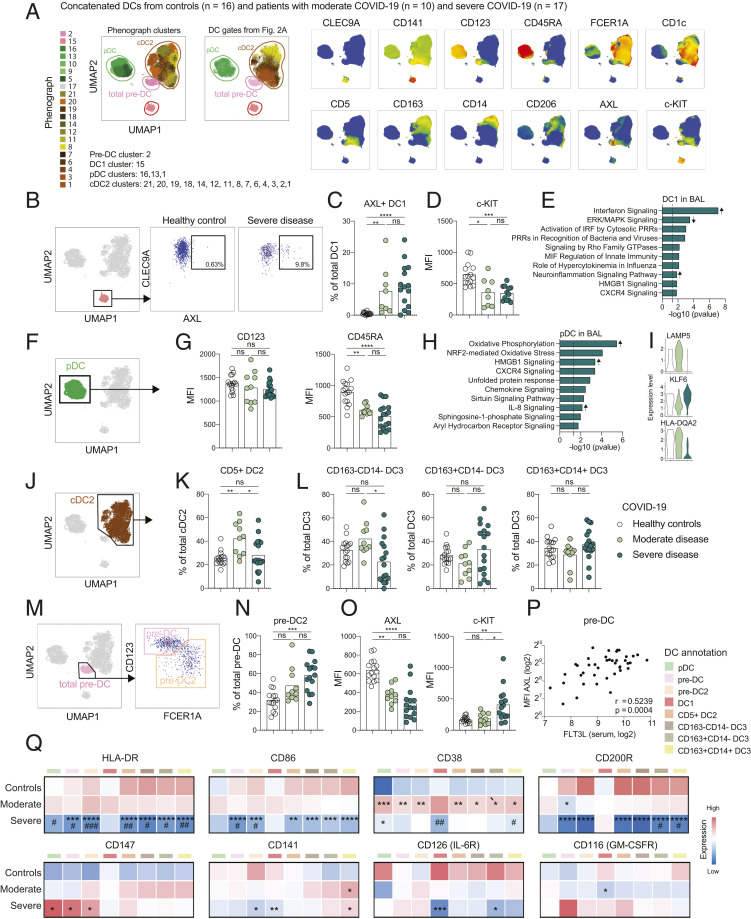
Altered activation and developmental phenotype of DCs in COVID-19. (*A*) All DCs from patients and controls concatenated in a UMAP, as described in Fig. 2*A*, with color-coded Phenograph clusters and manual DC gates; cell identity was established based on marker expression. (*B*) DC1s gated in a UMAP and subjected to analysis of percentage of AXL^+^ cells, representative healthy control and severe COVID-19 patient shown in a FACS plot. (*C*) Quantification of the percentage of AXL^+^ DC1s in the three cohorts. (*D*) MFI of c-KIT quantified in total DC1s, gated in DC UMAP as shown in *B*. (*E*) IPA of DEGs up-regulated in DC1s in BAL of severe COVID-19 patients compared to healthy controls; arrow indicate direction of up-regulation based on *z*-score; data reanalyzed from previously published report (22), as described in *Material and Methods*. (*F*) pDCs gated in a UMAP for downstream analysis. (*G*) MFI of CD123 and CD45RA quantified in total pDCs in the three cohorts. (*H*) IPA of DEGs up-regulated in pDCs in severe patients compared to moderate COVID-19; arrows indicate direction of up-regulation based on *z*-score; data reanalyzed from previously published report (22), as described in *Material and Methods*. (*I*) Genes in pDCs differentially expressed between severe and moderate COVID-19 patients, presented in the three cohorts; data reanalyzed from previously published report (22), as described in *Material and Methods*. (*J*) cDC2s gated in a UMAP for downstream analysis. (*K*) Quantification of the percentage of CD5^+^ DC2 among total cDC2s in the three cohorts. (*L*) Quantification of the percentage of the three DC3 subsets among total DC3s (as presented in Fig. 2*A*). DC3 subsets were defined from the cDC2 gate in *J* in the three cohorts. (*M*) Total pre-DCs gated in a UMAP subdivided in pre-DCs and pre-DC2s. (*N*) Quantification percentage of pre-DC2 in total pre-DCs. (*O*) MFI of AXL and c-KIT in total pre-DCs in the three cohorts. (*P*) Correlation between AXL MFI in pre-DCs and soluble FLT3L. (*Q*) Heatmap showing marker expression in the three cohorts, across the DC subsets, only samples with more than 10 cells included. Statistical evaluation was made separately in each indicated subset by comparing the MFI in the three cohorts; significance for healthy to moderate and healthy to severe comparisons is indicated by an asterisk (*) and for moderate to severe comparison is indicated by a pound sign (#). Statistical evaluation using Spearman test for correlation, Kruskal-Wallis test and Dunn’s multiple comparisons test for all other analysis. Samples with less than 10 cells in any of the DC subsets annotated in *Q* were excluded from the analyses. Significance level: *P < 0.05, **P < 0.01, ***P < 0.001, ****P < 0.0001; #*P* < 0.05, ##*P* < 0.01, ###*P* < 0.001.

Next, a detailed phenotypic mapping of each DC subset was performed. As a starting-point, Phenograph clustering was performed. The revealed clusters corresponded to all major DC subsets (Fig. 3*A*). When investigating the phenotype of DC1, a minor expansion of AXL^+^DC1, possibly related to type-I IFN signaling, was a general feature in COVID-19 along with decreased expression of the differentiation marker c-KIT (Fig. 3 *B*–*D*). Of note, IFN-signaling was also the most strongly activated pathway in DC1s at the site of infection as assessed in bronchioalveolar lavage (BAL) samples (see Fig. 3*E* for reanalysis of public sincle-cell RNA sequencing [scRNA-seq] data of BAL immune cells of COVID-19 patients and controls, and *SI Appendix*, Fig. S1). Circulating pDCs were observed with decreased levels of CD45RA (Fig. 3 *F* and *G*), while pDCs in BAL were enriched for cytokine and chemokine signaling pathways and further presented with reduced gene expression of *LAMP5*, known to be down-regulated in response to TLR9 stimulation and type I IFN signaling (28), in severe COVID-19 patients (Fig. 3*I*). Finally, higher levels of *KLF6*, a transcription factor induced by TGF-β in DCs (29), was increased in pDCs in BAL from severe COVID-19 patients, while the MHC class II molecule *HLA-DQA2* displayed lower transcript levels (Fig. 3*I*), in line with lower protein levels of HLA-DR detected on circulating pDCs.

Focusing on cDC2s, an increase in frequencies of CD5^+^ DC2s was detected in moderate COVID-19 (Fig. 3 *J* and *K*), while a reduction in frequencies of CD163^−^CD14^−^ DC3s was noted in severe COVID-19 with otherwise stable frequencies of the DC3 subsets (Fig. 3*L*). Analysis of the pre-DC subsets (Fig. 3*M*) showed a higher percentage of pre-DC2 among total pre-DCs was seen in severe COVID-19 (Fig. 3*N*). In pre-DCs, an up-regulation of the stem cell marker c-KIT was detected in severe COVID-19 (Fig. 3*O*), and AXL was decreased in response to infection. The levels of AXL correlated positively with serum levels of FMS-like tyrosine kinase 3 ligand (FLT3L), a factor central for DC development (Fig. 3*M*). Finally, levels of the maturation markers HLA-DR and CD86, the ecto-enzyme CD38, the inhibitory receptor CD200R, the GM-CSF receptor (CD116), the interleukin (IL)-6R (CD126), thrombomodulin (CD141), and a possible SARS-CoV-2 spike protein receptor CD147 were assessed across the DC subsets (Fig. 3*Q*). Higher levels of CD38 were detected specifically in moderately sick COVID-19 patients, while the maturation markers HLA-DR and CD86 were decreased over all DC subsets (with an exception for DC1s) in severe disease (Fig. 3*Q*). In severe disease, all subsets of the cDC2 linage presented with lower levels of CD200R, while DC1s specifically down-regulated the IL-6R (Fig. 3*Q*). Taken together, these analyses suggest lineage-specific changes and altered developmental phenotype in DC subsets from COVID-19 patients that occur either in response to SARS-CoV-2 infection or specifically in patients with severe COVID-19.

### Major Phenotypic Alterations within Monocyte Subpopulations of COVID-19 Patients.

**Fig. 4.**
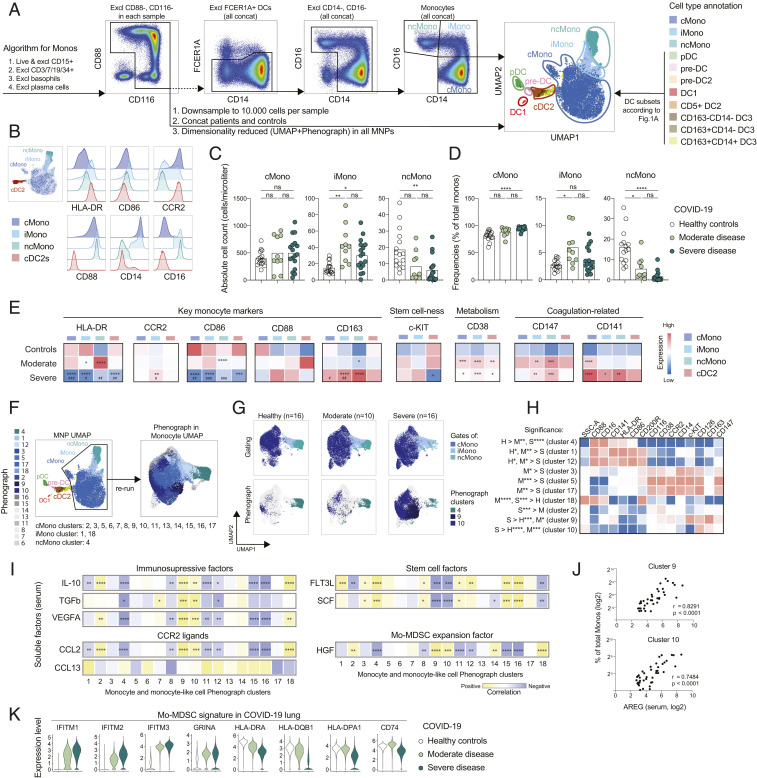
Major phenotypic alterations within monocytes and monocyte-like cells in COVID-19 patients. (*A*) Monocyte gating strategy projected together with DCs, gated as described in Fig. 2*A*, to the MNP UMAP. (*B*) Key monocyte markers in monocyte subsets, compared to cDC2s, in all concatenated samples. (*C*) Absolute numbers of monocytes in the three cohorts. (*D*) Monocyte frequencies in the three cohorts. (*E*) Heatmap showing marker expression for the three cohorts in the indicated monocyte subsets with cDC2s included as a reference, comparing expression level within each cell population individually. Statistical evaluation was made separately in each indicated subset by comparing the MFI in the three cohorts; significance for healthy to moderate and healthy to severe comparisons are indicated by an asterisk (*) and for moderate to severe comparison by a pound sign (#). (*F*) MNPs from patients and controls concatenated in a UMAP with color-coded manually gated cell subsets (*Left*), rerun on monocytes presented in a UMAP with color-coded Phenograph clusters (*Right*). (*G*) Color-coded manual gates (*Upper*) and indicated Phenograph clusters (*Lower*) in monocyte UMAP of concatenated samples. (*H*) Expression of markers in selected significantly differential Phenograph clusters among the three cohorts (see also *SI Appendix*, Fig. S2). (*I*) Heatmap of correlation between Phenograph clusters 1 to 18 and levels of soluble factors in serum, a gradient between yellow and blue indicates positive (yellow) and negative (blue) correlation. (*J*) Correlation between soluble serum AREG and percentage of Phenograph clusters 9 and 10 in total monocytes. (*K*) MDSC signature genes, calculated by comparing MDSC with monocytes in a previous report (34), in lung MNP compartment differentially expressed between severe COVID-19 patients and controls, presented in the three cohorts; data reanalyzed from previously published report (22), as described in *Material and Methods*. Statistical evaluation using Kruskal–Wallis test and Dunn’s multiple comparisons test for comparison between the three cohorts and Spearman test for correlations, “bimod” test for DEGs. Significance level: *P < 0.05, **P < 0.01, ***P < 0.001, ****P < 0.0001; #*P* < 0.05, ##*P* < 0.01, ###*P* < 0.001.

Next, we focused on monocytes and their subsets (Fig. 4*A*). As expected, three major populations of monocytes were identified based on CD14 and CD16 expression (i.e., CD14^+^CD16^−^ classical monocytes [cMonos], CD14^+^CD16^+^ intermediate monocytes [iMonos], and CD14^low^CD16^++^ nonclassical monocytes [ncMonos]) (Fig. 4*A*). UMAP-analysis verified the present gating strategy and demonstrated separation between monocytes and DCs (Fig. 4*A*). This was also the case for DC3s, which in many aspects are similar to monocytes in phenotype, especially due to their expression of CD14 (Fig. 4*A*). In accordance with previous publications, iMonos expressed the highest levels of HLA-DR while cMonos displayed relatively high levels of CCR2 (Fig. 4*B*). Assessment of absolute counts of monocyte populations in COVID-19 patients and controls revealed no change in cMono numbers, significantly increased iMono numbers in response to infection, especially in moderate patients, and declining numbers of ncMonos in all patients (Fig. 4*C*). A similar pattern was observed when monocyte subset frequencies were assessed (Fig. 4*D*). While increased CD38 expression across monocyte subsets was a general feature unrelated to disease severity, higher levels of CCR2 on iMonos, as well as lower levels of HLA-DR and CD86 with increased expression of CD163 in all monocyte subsets, were found in severe COVID-19 (Fig. 4*E*). Furthermore, CD141 expression was increased in severe COVID-19 in ncMonos and iMonos, while it was only increased in cMonos of moderately sick patients (Fig. 4*E*). In summary, this shows redistribution and an immature phenotype of the monocyte compartment in COVID-19 that is linked to disease severity.

### Dimensionality Reduction Reveals Myeloid-Derived Suppressor Cell Phenotype in Severe COVID-19.

To analyze the global monocyte landscape in an unbiased manner and integrate the contribution of all tested markers, we next reclustered all monocytes. This revealed high heterogeneity of cells that fell into the cMono category (Fig. 4*F*). Indeed, cMonos stratified into 15 Phenograph clusters, whereas only one or two clusters were found for iMono and ncMono (Fig. 4*F* and *SI Appendix*, Fig. S2*A*). When assessing these clusters in relation to disease severity, only one cluster (#4, corresponding to ncMonos) was reduced in all patients compared to controls, whereas clusters 9 and 10 were specifically increased in severe compared to moderately sick patients and healthy controls (Fig. 4*G* and *SI Appendix*, Fig. S2*A*). Next, when clusters with differential presence among the three groups (*SI Appendix*, Fig. S2*A*) were ordered sequentially (controls, moderate, and severe patients), a distinct phenotypic pattern emerged (Fig. 4*H*). In clusters enriched in moderate and severe patients, falling into the cMono category, higher expression of the GM-CSF receptor (CD116), IL-6R (CD126), CD147, CD38, and CCR2 was found. Monocyte clusters specific to severe COVID-19 patients (clusters 9 and 10), compared to the ones from patients with moderate disease (clusters 3, 5, and 17), had lower levels of HLA-DR and CD86 (Fig. 4*H*). This phenotype may be reminiscent of a monocytic myeloid-derived suppressor cell (Mo-MDSC)–like phenotype (30, 31). In addition, the Mo-MDSC–like cluster 9 expressed high levels of c-KIT and CD163 (Fig. 4*H*). Furthermore, a positive correlation was found between the cMono Phenograph clusters specific to severe COVID-19 (clusters 2, 9, 10, and 18) and the soluble immunosuppressive factors IL-10, TGF-β, and VEGFA, as well as with AREG (Fig. 4 *I* and *J*), the latter known to be involved in tolerance and tissue repair (32). In contrast, clusters 2, 9, 10, and 18 showed a negative association with stem cell factors important for myeloid cell development and differentiation (Fig. 4*I*). The Mo-MDSC–like clusters also correlated positively with hepatocyte growth factor (HGF), known to support Mo-MDSC expansion (33) (Fig. 4*I*).

To search for evidence of an Mo-MDSC signature at the site of infection, publicly available scRNA-seq data of BAL from healthy controls, moderate, and severe COVID-19 patients was used (22) (*SI Appendix*, Fig. S1 *A–P*). The expression of Mo-MDSC signature genes, calculated by comparing the transcriptional signatures of monocytes versus Mo-MDSC in a previous report (34), was significantly higher in MNPs from severe patients compared to controls (e.g., *IFITM1*, *IFITM2*, *GRINA*, *SOCS3*, and *CD84*) (Fig. 4*K* and Dataset S1). In line with the specific immune suppressive Mo-MDSC gene-expression pattern, MHC class II and *CD74* were found at lower levels in severe COVID-19 patients compared to controls (Fig. 4*K*). Altogether, this analysis revealed monocyte heterogeneity related to COVID-19 severity and an expansion of monocytes with a MDSC-like phenotype only observed in severe COVID-19 patients.

### Viremia and Seroconversion Are Associated with Recovery of Inflammatory DC3s.

**Fig. 5.**
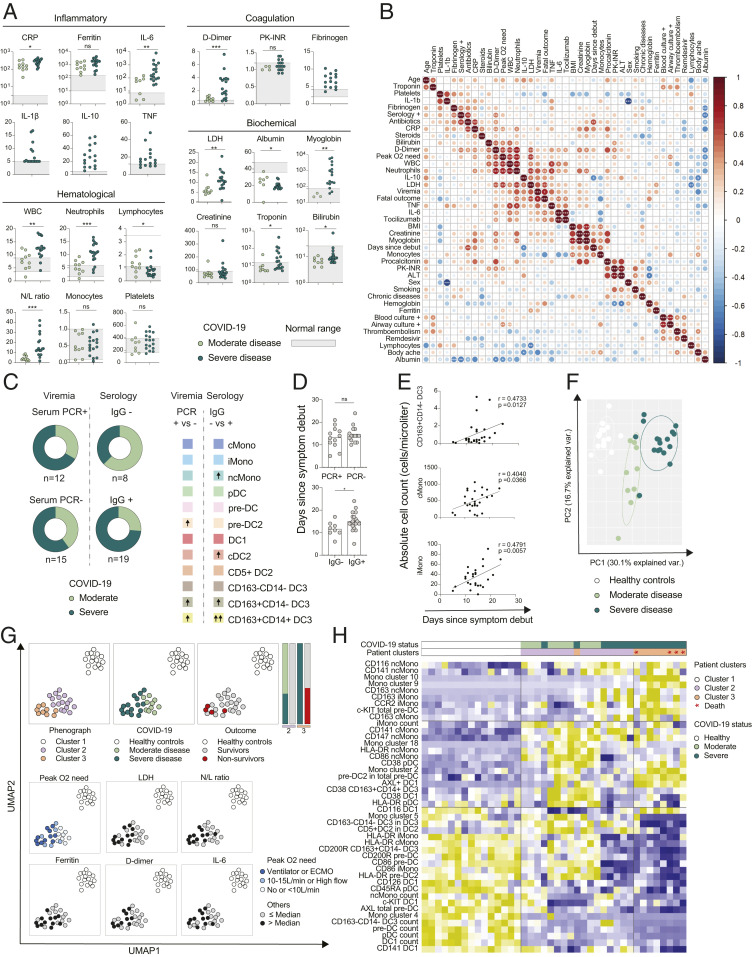
Clinical hyperinflammation, the MNP landscape, and COVID-19 outcome. (*A*) Levels of clinical analytes in moderate and severe COVID-19 patients, reference levels indicated as a gray shaded area; CRP, D-dimer in milligrams per liter; procalcitonin, ferritin, myoglobin in micrograms per liter; hemoglobin, albumin in grams per liter; WBC, neutrophils, lymphocytes, monocytes, platelets as 10^9^/L; troponin in nanograms per liter; fibrinogen in grams per liter; creatinine, bilirubin in micromoles per liter; LDH in units per liter; IL-6, IL-1β, TNF, IL-10 in nanograms per liter. (*B*) Integrated correlation clustering map of clinical parameters; the color of the circles indicated positive (red) and negative (blue) correlations, color intensity represented correlation strength as measured by the Pearson’s correlation coefficient. (*C*) Distribution of moderate and severe COVID-19 patients with respect to viremia (SARS-CoV-2 PCR^+/−^, measured at sampling) and serology (SARS-CoV-2 IgG^−/+^, measured at sampling) shown in donut charts (*Left*); differences in absolute numbers of MNP populations between SARS-CoV-2 PCR^+^ and SARS-CoV-2 PCR^−^ patients, and between SARS-CoV-2 IgG^−^ and SARS-CoV-2 IgG^+^ patients (*Right*), upward arrows indicate significant increase (*SI Appendix*, Fig. S3*B*). (*D*) Differences in days since symptom debut until sample collection in SARS-CoV-2 PCR^+/−^ and SARS-CoV-2 IgG^−/+^ patients. (*E*) Correlation between days since symptom debut until the sample collection and absolute numbers of cells within MNP populations (*SI Appendix*, Fig. S3*C*). (*F*) PCA of 108 MNP parameters in the three cohorts (*SI Appendix*, Fig. S3*D*). (*G*) Dimensionality reduction of 108 MNP parameters for each patient or control presented in UMAP color-coded annotated by Phenograph clusters, disease status, and outcome (above dashed line), as well as levels of clinical parameters; that is, peak O_2_ need, LDH, neutrophil/lymphocyte (N/L) ratio, D-dimer, ferritin, and IL-6, converted to categorical variables based on a median value (below dashed line). (*H*) Hierarchical clustering of patients from the three Phenograph clusters and 44 selected preclinical parameters. Statistical evaluation using Mann–Whitney *U* for comparison between the two groups, Spearman test for correlations of nonnormally distributed data and Pearson test for correlations normally distributed data. Significance level: *P < 0.05, **P < 0.01, ***P < 0.001.

From a clinical point of view, all patients presented with elevated inflammatory markers though clinical hyperinflammation and signs of coagulation disturbances were most pronounced in the severe COVID-19 patients (Fig. 5*A* and *SI Appendix*, *Material and Methods*). Of note, the laboratory measurements and reference values for the clinical parameters were obtained from the established clinical routine laboratory assays at the Karolinska University Hospital Laboratory (Fig. 5 *A* and *B*). Integrative correlation mapping of clinical parameters, obtained from the routine monitoring regimen for hospitalized COVID-19 patients—including peak measurements recorded during hospitalization up until study sampling (Fig. 5*B*), and those taken within 24 h from MNP profiling (*SI Appendix*, Fig. S3*A*)—revealed that levels of inflammatory cytokines and LDH clustered together with fatal outcome and viremia. Indeed, subgroups of moderate and severe COVID-19 patients had detectable virus in serum (serum PCR^+^, measured at study sampling), while the majority of seroconverted (SARS-CoV-2 IgG^+^, measured at study sampling) patients were critically ill (74%, 14 of 19) (Fig. 5*C*). Significantly higher numbers of pre-DC2 as well as of inflammatory DC3 (CD163^+^CD14^+^ DC3 and CD163^+^CD14^−^ DC3) were found in patients without ongoing viremia (serum PCR^−^) and the same DC3 populations were elevated in seroconverted patients (Fig. 5*C* and *SI Appendix*, Fig. S3*B*). Following a similar trend, ncMonos were also present at higher numbers in seroconverted patients (Fig. 5*C* and *SI Appendix*, Fig. S3*B*). As expected, patients that had seroconverted (SARS-CoV-2 IgG^+^) were sampled slightly later in their disease course (Fig. 5*D*). Finally, absolute numbers of the inflammatory DC3 subset (CD163^+^CD14^−^ DC3), as well as iMonos and cMonos, increased over time after onset of symptoms (Fig. 5*E* and *SI Appendix*, Fig. S3*C*), suggesting that seroconverted patients had passed the time-point when their DC3s were at their lowest levels. Thus, subset-specific changes in absolute numbers of MNPs depended on the time from symptom onset, and a time-dependent recovery of DC3 and an increase of monocytes and monocyte-like cells were noted.

### Independent Assessment of the MNP Landscape Identifies Clinical Subgroups and Disease Outcome-Correlates in COVID-19.

To get an overall view of the MNP landscape in relation to COVID-19 severity, we performed a principal component analysis (PCA) of the 108 MNP parameters that had been measured. COVID-19 patients clearly separated from healthy controls, and within the patients, those with moderate and severe disease further clustered apart (Fig. 5*F*). The MNP parameters that drove the separation between the groups were: Higher DC counts and Mono cluster 4 (ncMonos), higher c-KIT expression in DC1, higher CD45RA expression in pDCs, and higher HLA-DR, CD86, and CD200R expression in cDC2 lineage in controls; higher CD38 expression in MNPs, higher frequency of CD5^+^ DC2s among cDC2s in moderate patients COVID-19; and an expansion of Mo-MDSC-like clusters (9 and 10), higher c-KIT levels in DC progenitors, and higher frequencies of pre-DC2s in severe patients (Fig. 5*F* and *SI Appendix*, Fig. S3*D*). Dimensionality reduction using UMAP and Phenograph was performed using the same MNP variables, revealing three major clusters, where cluster 1 corresponded to healthy controls, cluster 2 corresponded to all moderate and some severe COVID-19 patients, and cluster 3 corresponded to severe COVID-19 patients only (Fig. 5*G*). All patients with fatal outcome were found in cluster 3 and these further had a high oxygen requirement, exhibited higher levels of LDH (peak measurement), a high neutrophil/lymphocyte ratio, and elevated ferritin (measured within 24 h from MNP profiling) (Fig. 5*G* and *SI Appendix*, Fig. S3*E*).

To visualize the key MNP profile components specific for each cluster, 44 of the most significant MNP parameters analyzed so far were selected and subjected to hierarchical clustering. Monocyte clusters 9 and 10, corresponding to Mo-MDSC–like cells, were specific to patient cluster 3 with the nonsurvivors. These parameters also clustered together with CD163 expression on all monocyte subsets, CCR2 expression on iMonos, and c-KIT in total pre-DCs. For moderate disease, higher expression of CD38 in all DC lineages, CD141 on cMono, and higher levels of monocyte cluster 18 (iMono, the only cluster expressing high levels of CD200R among the patient-specific clusters), were the determining parameters. A set of markers were lost in severe patients, including HLA-DR and CD86 in both monocytes and DCs, and CD200R in DC subsets (Fig. 5*H*). Moreover, the CD200R decrease in nonsurvivors was restricted to the cDC2 lineage, with the most significant differences seen in progenitors (pre-DCs and pre-DC2) (*SI Appendix*, Fig. S3*F*). To test whether this was entirely dependent on severity status rather than outcome, CD200R levels were assessed within severe patients only. A similar pattern was observed, suggesting that CD200R is diminished in the cDC2 lineage and is related not only to disease severity but also to fatal outcome (*SI Appendix*, Fig. S3*F*). Altogether our results suggest lineage restricted phenotypic and developmental shifts in MNPs and their late precursors and show that MNPs, alone, could identify a cluster of COVID-19 nonsurvivors.

## Discussion

**Fig. 6.**
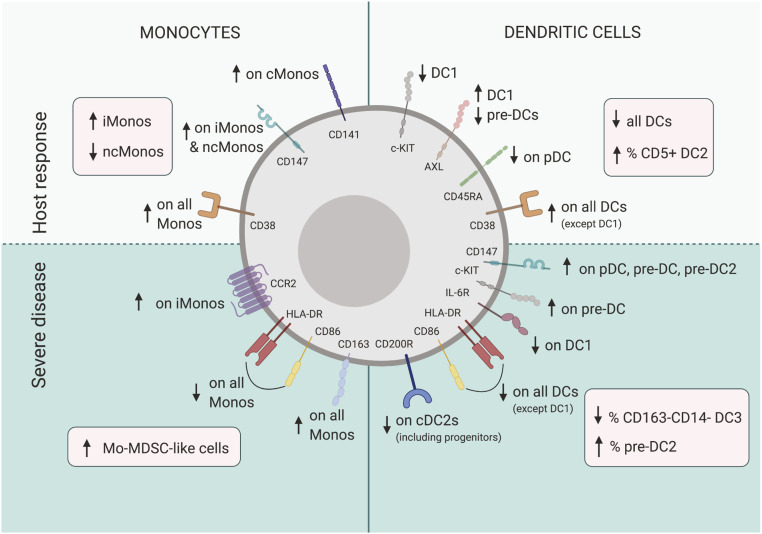
Summary of MNP response to SARS-CoV-2 and in relation to COVID-19 severity. Monocytes (*Left*) and dendritic cells (*Right*), respond to SARS-CoV-2 (*Upper*) with decreased circulating levels of all DC subsets, but higher CD5^+^ DC2 frequency, activated phenotype (up-regulation of ecto-enzyme CD38, expansion of CD14^+^CD16^+^ iMonos, decrease of CD14^low^CD16^++^ ncMonos, and lower CD45RA levels on pDCs), and features pointing to viral sensing and type I IFN imprint found in DC1s (e.g., lower levels of c-KIT, expansion of AXL^+^DC1s). Severe COVID-19 (*Lower*) is associated with altered developmental DC phenotype (e.g., increased frequencies of late pre-DC2 progenitors and increased stem cell marker c-KIT expression in pre-DCs, as well as immature phenotype on all DC subsets with lower HLA-DR and CD86), expansion of Mo-MDSC–like cells and decrease in inhibitory receptor CD200R, specific to cDC2 lineage. Arrows in red boxes indicate changes in counts and frequencies of cell populations. Arrows in green areas illustrate altered expression of receptors within indicated populations.

While broad efforts toward an overview of immune cell- and soluble-factor alterations in COVID-19 are under way, a deep and comprehensive understanding of the MNP system, including circulating progenitors, is still largely lacking. Here, we used high-dimensional flow cytometry to characterize the MNP landscape in well-defined prospective cohorts of SARS-CoV-2–infected patients with moderate and severe COVID-19, as well as matched SARS-CoV-2 IgG^−^ healthy controls. This allowed a robust mapping of phenotypic and developmental alterations in circulating DCs and monocytes that occurred either in response to clinical infection or specifically in severely sick patients (Fig. 6). By combining conventional gating with unsupervised dimensionality reduction, we provide a reference for absolute numbers and lineage-dependent phenotypic shifts in MNP subsets in response to SARS-CoV-2. This analysis revealed an affected developmental phenotype in the cDC2 lineage and expansion of monocytic MDSC-like cells in critically ill patients. In addition, the results were related to data from the site of infection and severe inflammatory impact by means of reanalyzing BAL scRNA-seq data and relevant soluble factors in circulation. Finally, and strikingly, alterations within the MNP landscape alone were found to separate healthy controls from patients, and to further identify distinct patient clusters, such as moderate and severe as well as within severe patients even those with fatal outcome. The present high-dimensional mapping of the MNP landscape yields insights into the MNP response during SARS-CoV-2 infection and in relation to COVID-19 severity and provides a comprehensive framework for future studies (Fig. 6).

Knowledge on alterations of specific DC subsets and their circulating progenitors in acute viral infection in humans is limited. However, the depletion of all DCs and their precursors detected here is in line with previous studies showing DC disappearance in circulation during acute viral insults (20, 35, 36). Given their central role in antigen recognition and control of adaptive immunity (37), as well as their presence in COVID-19 lung (22), it is plausible that DC disappearance from the circulation is related to their influx to tissues and lymph nodes to lead and assist initiation of adaptive immune responses aiming at virus elimination. In support of this, we find recovery of absolute numbers of pre-DC2s, as well as subsets of DC3s in patients where virus has been cleared from circulation and seroconversion occurred. Other features of host response included decreased levels of c-KIT in DC1, expansion of AXL^+^ DC1 and CD5^+^ DC2s, similarly to monocytes increased expression of the ecto-enzyme CD38 in most DC subsets, and decreased CD45RA on pDC, possibly indicating activation as also detected in lung tissue. It has been shown that DC1s down-regulate c-KIT upon viral stimulation in an IFN-dependent manner (38), and it is thus possible that lower c-KIT levels in DC1 found here might indicate DC1 SARS-CoV-2 sensing. In support of this, pathways analysis of DC1s in COVID-19 lung revealed pattern-recognition receptor-related pathways and an IFN-response imprint. Although minor, the expansion of AXL^+^ DC1 in circulation could further support an IFN-affected phenotype, as AXL has been shown to be up-regulated on myeloid cells upon type I IFN stimulation (39). However, even if supported by transcriptional data of DC1s from BAL of COVID-19 patients, our observation indicating the occurrence of an IFN imprint in DC1s merits functional exploration in future studies.

In severe COVID-19, the DC phenotype showed affected maturation status with lower levels of HLA-DR and CD86 in all DC subsets, with an exception for DC1, and an altered developmental phenotype with expansion of late progenitors pre-DC2 and increased expression of the stem cell marker c-KIT in pre-DCs. Additional lineage-specific DC alterations in severe COVID-19 included down-regulation of IL-6R in DC1 and a drastic decrease of the inhibitory receptor CD200R in the cDC2 lineage, including circulating progenitors. Decreased CD200R levels are of particular interest in development of severe COVID-19, given that CD200R signaling is known to modulate immune responses to pathogenic stimuli, control myeloid cell function, inhibit proinflammatory cytokine expression, and inhibit tissue damage caused by myeloid-derived cells (40, 41). Moreover, it has been suggested that CD200R signaling plays an important role in T cell priming during viral infection (42), but remains to be studied in further detail in COVID-19. Our findings suggest that in severe COVID-19, DCs, and in particular the cDC2 lineage, are immature with alterations in the developmental phenotype, lack up-regulation of the activation marker CD38, as seen in moderate patients, and lose the inhibitory receptor CD200R, all possibly contributing to inefficient regulation of proinflammatory conditions in severe COVID-19.

In line with previous reports, we observed an expansion of intermediate CD14^+^CD16^+^ monocytes in COVID-19 patients (21, 43). This appeared to be a general feature of clinical SARS-CoV-2 infection. In addition, all monocyte subsets responded with increased CD38 expression that, in contrast to DCs, was independent of disease severity. It is plausible that iMonos, exhibiting a strong IFN signature in COVID-19 (27), contribute to successful viral clearance in moderate COVID-19 patients. In addition, the Phenograph cluster we identified that corresponded to iMonos (cluster 18) (Fig. 4*H*) had high levels of the inhibitory receptor CD200R, supporting the hypothesis that robust innate antiviral responses as well as efficient control of the proinflammatory state are important in order to have a moderate course of COVID-19 (44). On the other hand, in line with previous observations (19, 27, 45), monocytic cell clusters with an immature phenotype (in particular cluster 9 and 10), identified as Mo-MDSC–like cells, were linked to severe disease and a patient cluster that included all nonsurvivors. In addition, a positive correlation between Mo-MDSC and HGF, known to mediate Mo-MDSC expansion (33), was observed (Fig. 4*I*). As severe dysregulation of myelopoiesis has been reported in COVID-19 (27), Mo-MDSC–like cells detected here could represent immature forms of MNPs with or without suppressive functions. Further studies are required to understand whether Mo-MDSC–like cells described here in fact have suppressor functions (e.g., through a supply of immunosuppressive cytokines) that in the present study showed a positive correlation with Mo-MDSC–like cell frequencies. In support of expansion of functional Mo-MDSC in severe COVID-19, it has been proposed that expanded Mo-MDSC isolated from COVID-19 patients suppressed T cell proliferation and IFN-γ production partly via an arginase-1–dependent mechanism (46). In line with previous observations in COVID-19 (20, 27, 36, 43) and other infectious and inflammatory settings (35, 47–49), loss of ncMonos was detected among circulating monocytes, a pattern that was mostly evident in severe COVID-19 patients.

Focusing on MNP biology in COVID-19, our study has limitations to be considered. First, the present study has a relatively small sample size, and thus lacks power to draw firm conclusions on clinical variables, such as outcome or biomarkers predicting certain clinical courses. However, it is powered and designed to interpret phenotypic changes within the MNP system in relation to COVID-19 severity. It is also realized that the addition of a disease-control group (e.g., non–COVID-19 patients with severe infections or inflammatory conditions) could have further strengthened the conclusions drawn. Thus, the alterations noted in the MNP landscape of COVID-19 patients might at varying degrees be shared with other febrile illnesses. Finally, since we largely excluded the oldest patients, those with ongoing malignancies, and patients receiving immune-suppressive treatment prior to admission in order to study a more homogeneous patient cohort, future larger studies should also assess the impact of age and underlying conditions on the MNP response in COVID-19.

Hyperinflammation in COVID-19 is characterized by high systemic cytokine levels combined with coagulation abnormalities and thromboembolism (2, 50, 51), also indicated in our study by increased levels of fibrinogen and D-dimer. Increased levels of thrombomodulin (CD141), a cofactor for thrombin reducing blood coagulation, possibly possessing an anticoagulant potential, were observed on cMonos in both moderate and severe disease, and on iMonos and ncMonos in severe COVID-19. While the role of the extracellular metalloprotease inducer CD147 that was discussed to be a viral spike protein receptor remains to be determined, it is also a potential host factor in infection-mediated coagulation (52). In the present study, elevated levels of CD147 on iMonos and ncMonos was a general feature of all patients, while increased levels of CD147 on pDCs, pre-DCs, and pre-DC2s were specific to severe COVID-19. In this context, it is interesting to note that DC progenitors can themselves be susceptible to viral insult (53). It remains to be addressed what causes CD147 up-regulation in COVID-19 and how the MNP system may participate in dysregulated coagulation, but it has previously been shown that CD147 supports platelet–monocyte interactions promoting vascular inflammation (54). In addition, CD147 is up-regulated upon TNF and IFN-γ stimulation, an effect strongly potentiated by bacterial stimuli (55). Of note, the two pulmonary embolism cases in our cohort occurred in severe COVID-19 patients suffering from superinfection with positive blood cultures.

In summary, we here provide a comprehensive mapping of the MNP landscape in COVID-19. Lineage-restricted changes were observed with loss of the inhibitory receptor CD200R in cDC2s, including the progenitors, associated with severe disease and fatal outcome as well as expansion of immature Mo-MDSC–like monocytes in the critically ill. These findings suggest dysregulation of myeloid development in severe COVID-19, involving both monocyte and DC lineages, and changes detected in circulation were mirrored in lung tissue. Unsupervised clustering analysis revealed that MNPs, alone, could identify a cluster with nonsurvivors. Further research is needed to understand if and how immature myeloid derived cells in COVID-19 contribute to perpetuation and silencing of the inflammatory storm, and how the altered MNP development affects monocyte and DC ability to contribute to viral clearance. The knowledge gained from investigating MNP biology provides a framework that has the potential to influence vaccine development, where accurate MNP host responses are needed, and improvement of treatment strategies aiming at controlling the proinflammatory storm without affecting the immune system’s ability to clear the virus.

## Materials and Methods

### Patient and Healthy Control Cohorts.

COVID-19 patients were recruited at the Karolinska University Hospital in Stockholm. All patients were SARS-CoV-2 PCR^+^ in nasopharynx or sputum, had clinical COVID-19, and were sampled within 8 d from hospitalization. Moderate COVID-19 patients and an oxygen saturation (SO_2_) between 90% and 94% or had a 0.5 to 3 L/min oxygen requirement at screening. Severe COVID-19 patients were treated at the intensive care unit or a high-dependency unit. Patients with an ongoing malignancy or ongoing immune-suppressive medication prior to hospitalization were excluded from the study. The patients were further described by Sequential Organ Failure Assessment score (56) and the NIH ordinal scale (57). The NIH ordinal scale scores are as follows: 1, not hospitalized, no limitations; 2, not hospitalized, with limitations; 3, hospitalized, no active medical problems; 4, hospitalized not on oxygen but requiring ongoing medical care; 5, hospitalized, on oxygen; 6, hospitalized, on high-flow oxygen or noninvasive mechanical ventilation; 7, hospitalized, on mechanical ventilation or extracorporeal membrane oxygenation; 8, death. In addition, a group of healthy volunteers matched for age and sex, no history of COVID-19, no signs or symptoms of ongoing COVID-19, and SARS-CoV-2 IgG seronegative, was recruited in parallel as a control group. The research was approved by the Swedish Ethical Review Authority, and all participants provided informed consent.

### Serology.

SARS-CoV-2 IgG titers were measured at the day of sampling in serum in all the patients and controls according to the routinely used clinical pipeline at the Clinical Microbiology Department, Karolinska University Hospital, and finally interpreted by a clinical microbiologist as positive or negative, according to clinical practice.

### Viral Titers and Clinical Blood Tests.

Viral load was assessed at the day of sampling as serum SARS-CoV-2 PCR positivity, examined in serum from all the patients following the pipeline at the Clinical Microbiology Department, Karolinska University Hospital. Briefly, two independent tests were used and finally interpreted by a clinical microbiologist as positive or negative, according to the clinical routine. Clinical laboratory blood tests were performed at the Karolinska University Laboratory as a part of clinical routine monitoring of COVID-19 patients.

### Trucount Staining.

In order to determine the absolute leukocyte numbers, trucount staining was performed. Briefly, 50 µL EDTA blood and 20 µL of antibody mix (6-color TBNK Reagent and CD123 BUV395 from BD, CD15 PB, CD193 BV605 and HLA-DR BV785 from Biolegend, and CD14 PE-Cy5 from eBioscience) were added in BD trucount tubes and incubated for 15 min at room temperature in the dark. The samples were then fixed using 430 µL 1× BD FACS lysing solution (BD Biosciences).

### Sample Processing and Flow Cytometry.

Peripheral blood mononuclear cells (PBMCs) were isolated from patient and control blood samples after Ficoll separation using Lymphoprep (Stemcell Technologies) and FACS staining was performed on freshly isolated cells in three independent experiments during three subsequent weeks to minimize batch effects. To assess batch-related effects, internal control of frozen healthy PBMCs were used in all experiments and revealed minimal variability. Briefly, PBMCs were resuspended in PBS containing 2% FCS and 2 mM EDTA and a mixture of antibodies, supplemented with BD Horizon Brilliant Stain Buffer Plus (BD Biosciences) at 1:5 and FcR Blocking Reagent (Miltenyi Biotec) at 1:25, and were incubated for 30 min. Next, cells were washed twice and fixed in PBS containing 1% PFA for 2 h in order to inactivate the virus. Cells were acquired on a FACSymphony A5 instrument (BD Biosciences), equipped with UV (355 nm), violet (405 nm), blue (488 nm), yellow/green (561 nm), and red laser (637 nm). Details of antibodies used as well as information on filters for cytometer configuration are provided in *SI Appendix*, Table S3.

### Flow Cytometry Analysis.

Acquired data were analyzed using DIVA (BD Biosciences), FlowJo v.10.5.3 (BD Biosciences), and Prism v8.0.2 (GraphPad Software). Absolute numbers of MNPs were determined relating to CD14^+^ cells in the trucount staining. Algorithms used for dimensionality reduction were UMAP (58) (https://github.com/lmcinnes/umap) and Phenograph (59) (https://github.com/JinmiaoChenLab/Rphenograph). UMAP coordinates and Phenograph cluster annotation were assigned to each cell in each sample in the concatenated sample file, and from there, subset-specific phenotypic changes of mean fluorescence intensity (MFI) were analyzed by gating directly in the UMAP space on the concatenated samples using FlowJo v.10.5.3 (BD Biosciences).

### Soluble Factor Analysis.

Serum levels of soluble factors were analyzed at the day of the sampling using proximity extension assay, based on real-time PCR quantification of pair-wise binding of oligonucleotide-labeled target antibodies (Olink Proteomics; sensitivity, specificity, dynamic range, repeatability, reproducibility, and scalability validation documents are available at https://www.olink.com/resources-support/document-download-center/).

### Single Cell Analysis.

Seurat v3 was used to reanalyze single cell data. In brief, scRNA-seq from a previously published report (22) was used and 10X Genomics filtered_feature_bc_matrix files were acquired from Gene Expression Omnibus (GEO; accession no. GSE145926). Quality control, such as gene number between 200 and 6,000, UMI count above 1,000, and mitochondrial gene percentage below 0.1 were applied, and “LogNormalize” method in Seurat v3 was used to normalize the data matrix followed by PCA using the top 2,000 most variable genes. The top 50 principal components were used to visualize the cells in UMAP, and a “bimod” test was used to detect differentially expressed genes (DEGs). First, we selected myeloid DC clusters 22, 25, 27 (CLEC9A, CADM1, CD1c, FCER1A), pDC cluster 28 (LILRA4, IL3RA), and other myeloid clusters 0, 1, 2, 3, 4, 5, 6, 9, 10, 11, 12, 13, 21, 23, 29 (CD14, CD68), after exclusion of B cells, plasma cells, epithelium, NK cells, and T cells (*SI Appendix*, Fig. S1 *A*–*F*). After rerunning total myeloid cells through the dimensionality reduction pipeline (*SI Appendix*, Fig. S1 *G*–*K*), neutrophils were excluded (*SI Appendix*, Fig. S1 *F* and *G*). To identify DCs, DC-containing clusters 11, 27, and 29 from the myeloid cell UMAP were reclustered (*SI Appendix*, Fig. S1 *L*–*P*). As cluster 4 contained a comparable number of cells in severe and moderate cohorts, and cluster 7 in severe and healthy, the DEGs in those clusters were calculated between the severe patients and the respective cohort and subjected to pathway analysis. Ingenuity Pathway Analysis (IPA) was performed to study pathways (Content version: 51963813; Release date: 2020-03-11; Ingenuity Systems). Predicted up-regulation and down-regulation of pathways was based on *z*-score, where a positive score implied up-regulation and a negative score implied down-regulation.

### PCA and Correlation Plots.

PCA plots were created using “prcomp” function and “ggbiplot” package in R and correlation mapping was performed using the “corrplot” package in R. The color of the circles indicated positive (red) and negative (blue) correlations; color intensity represented correlation strength as measured by the Pearson’s correlation coefficient. The correlation matrix was reordered using “hclust” for hierarchical clustering order. Significance tests were performed to produce *P* values and confidence intervals for each pair of input features.

### Statistical Analysis.

Differences between two groups were evaluated using a Mann–Whitney *U* test, and Kruskal–Wallis test with Dunn’s multiple comparisons test were used to evaluate differences among the three groups in all the analysis. Friedman test was used in Fig. 2*E* to evaluate differences among paired variables. A Spearman test was used for correlations of nonnormally distributed data and Pearson test was used for normally distributed data. Significance for pathways analyses in Fig. 3 *E* and *H* was defined by the IPA software (Ingenuity Systems). Significance level: *P < 0.05, **P < 0.01, ***P < 0.001, ****P < 0.0001.

## Supplementary Material

Supplementary File

Supplementary File

## Data Availability

Curated flow cytometry data will be made available for exploration via the Karolinska KI/K COVID-19 Immune Atlas homepage (https://covid19cellatlas.com) or from the corresponding author (E.K.) upon request.
