# Supplementary material for: Major alterations in the mononuclear phagocyte landscape associated with COVID-19 severity
Source: Proc Natl Acad Sci U S A. 2021 Jan 21;118(6):e2018587118. doi: 10.1073/pnas.2018587118 (PMC8017719; doi:10.1073/pnas.2018587118)
Supplement: Supplementary File [file pnas.2018587118.sapp.pdf]

Major alterations in the mononuclear phagocyte landscape associated with COVID-19 severity

Egle Kvedaraite*, Laura Hertwig, Indranil Sinha, Andrea Ponzetta, Ida Hed Myrberg, Magda Lourda, Majda Dzidic, Mira Akber, Jonas Klingström, Elin Folkesson, Jagadeeswara Rao Muvva, Puran Chen, Sara Gredmark-Russ, Susanna Brighenti, Anna Norrby-Teglund, Lars I. Eriksson, Olav Rooyackers, Soo Aleman, Kristoffer Strålin, Hans-Gustaf Ljunggren, Florent Ginhoux, Niklas K. Björkström, Jan-Inge Henter, Mattias Svensson, Karolinska KI/K COVID-19 Study Group

* Corresponding author: egle.kvedaraite@ki.se

SI Appendix table of contents:

1. Material and Methods
2. Fig. S1
3. Fig. S2
4. Fig. S3
5. Table S1
6. Table S2
7. Table S3
8. Dataset S1: DEGs in lung MNPs, related to Fig. 3, Fig. 4 and SI Appendix, Fig. S1 (separate .xlsx file)
9. Members of Karolinska KI/K COVID-19 Study Group

MATERIAL AND METHODS

Detailed cohort description

To address general features of the clinical biomarker pattern of COVID-19 we investigated parameters measured in the clinic as a part of routine monitoring, including inflammatory, hematological, biochemical and coagulation related parameters, and compared them between the two cohorts of COVID-19 patients, reference values indicated in grey (Fig. 5A). As expected, almost all patients had elevated levels of almost all inflammatory parameters previously associated with COVID-19, such as CRP, ferritin, as well as the cytokine levels (IL-10, IL-6, IL-1β, TNF), and differences in IL-6 levels was detected between the two study cohorts with higher levels in the critically ill patients. Coagulation parameters were also affected, and higher levels of D-dimer were detected in the severe patients, who also had elevated levels of fibrinogen (85%, 11/13). Biochemical status revealed higher levels of lactate dehydrogenase (LDH) and myoglobin in critically ill patients and hypoalbuminemia in all, with lower levels in severe disease. From the hematological perspective, severe patients had higher levels of WBC, higher neutrophil counts, lower lymphocytes and subsequently higher neutrophil to lymphocyte ratio, with no striking differences in platelets or monocytes (Fig. 5A). Radiologically, all patients examined showed bilateral infiltrates on chest x-ray; therapeutically, the majority received anticoagulants (93%, 25/27) and more than half broad-spectrum antibiotics (56%, 15/27). From a HLH perspective, all patients had fever at admission (100%, 27/27) and elevated ferritin levels (100%, 26/26, data not available for 1 patient), but no suspicion from the hematological parameters available, and in line rather elevated than low fibrinogen levels. Four patients in the severe group had superinfections (24% of severe patients, 4/17), in two of whom pulmonary embolism was detected, representing all detected cases of pulmonary embolism. To further address clinical laboratory status in relation to each other clinical parameters and outcome, integrative correlation mapping was performed (Fig. 5B).

This showed cytokines (IL-10, TNF, IL-6), LDH, neutrophil and D-dimer levels clustering together with fatal outcome, peak oxygen need and viremia. Of note, levels of monocytes showed a positive correlation with body mass index (BMI), a known COVID-19 risk factor, and monocytes were the only clinically available cell type correlating with days from symptom debut, indicating the myeloid mononuclear cell importance in disease development timeline. We also investigated symptom panorama at admission, and found that in addition to fever, all patients had dyspnea (100%, 27/27), most had cough (85%, 23/27), few had GI related symptoms (15%, 4/27), while body ache showed higher variation (44%, 12/27), and correlated with thromboembolism, days since symptom debut and, intriguingly, levels of monocytes (Fig. 5B). Importantly, a similar pattern was observed when integrative correlation mapping was performed including clinical parameters available 24 hours within MNP profiling (SI Appendix, Fig. S3A). Further details on clinical and laboratory findings are presented in SI Appendix Tables S1 and S2.

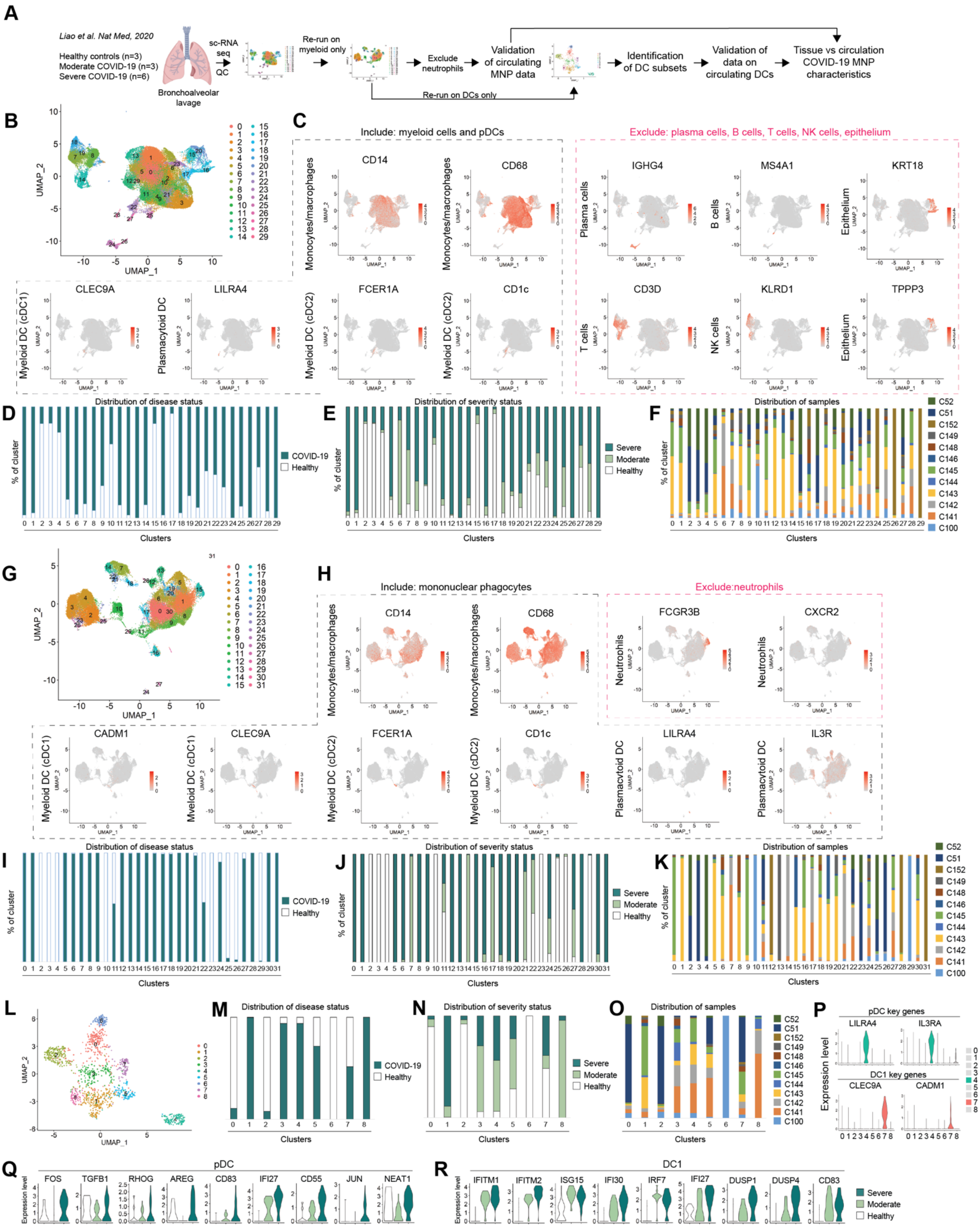

Fig. S1. Sc-RNAseq re-analysis (related to Fig. 3, Fig. 4, and Methods section "Single cell analysis")

(A) Analytic pipeline of sc-RNAseq data. (B) After quality control, scRNA-seq data re-analyzed using the Seurat version 3 (V3). (C) Expression plots of key markers used to discriminate between cells to be included and excluded in subsequent analysis. (D) Distribution of cells from COVID-19 patients and healthy controls among clusters generated in the whole data set. (E) Distribution of cells from moderate and severe COVID-19 patients and healthy controls among clusters generated in the whole data set. (F) Distribution of cells from each sample among clusters generated in the whole data set. (G) Selected myeloid cell clusters re-analyzed. (H) Expression plots of key markers used to discriminate between MNP and neutrophil clusters. (I) Distribution of cells from COVID-19 patients and healthy controls in MNP clusters. (J) Distribution of cells from moderate and severe COVID-19 patients and healthy controls in MNP clusters. (K) Distribution of cells from each sample in MNP clusters. (L) Clusters containing DCs selected and re-analyzed. (M) Distribution of cells from COVID-19 patients and healthy controls in DC clusters. (N) Distribution of cells from moderate and severe COVID-19 patients and healthy controls in DC clusters. (O) Distribution of cells from each sample in DC clusters. (P) Key genes of pDC and DC1 in cluster 4 (green) and 7 (red), respectively. (Q) Genes in pDCs differentially expressed between severe and moderate COVID-19 patients, presented in the three cohorts. (R) Genes in DC1s differentially expressed between severe COVID-19 and healthy controls, presented in the three cohorts. Statistical evaluation using ‘bimod’ test for differentially expressed genes.

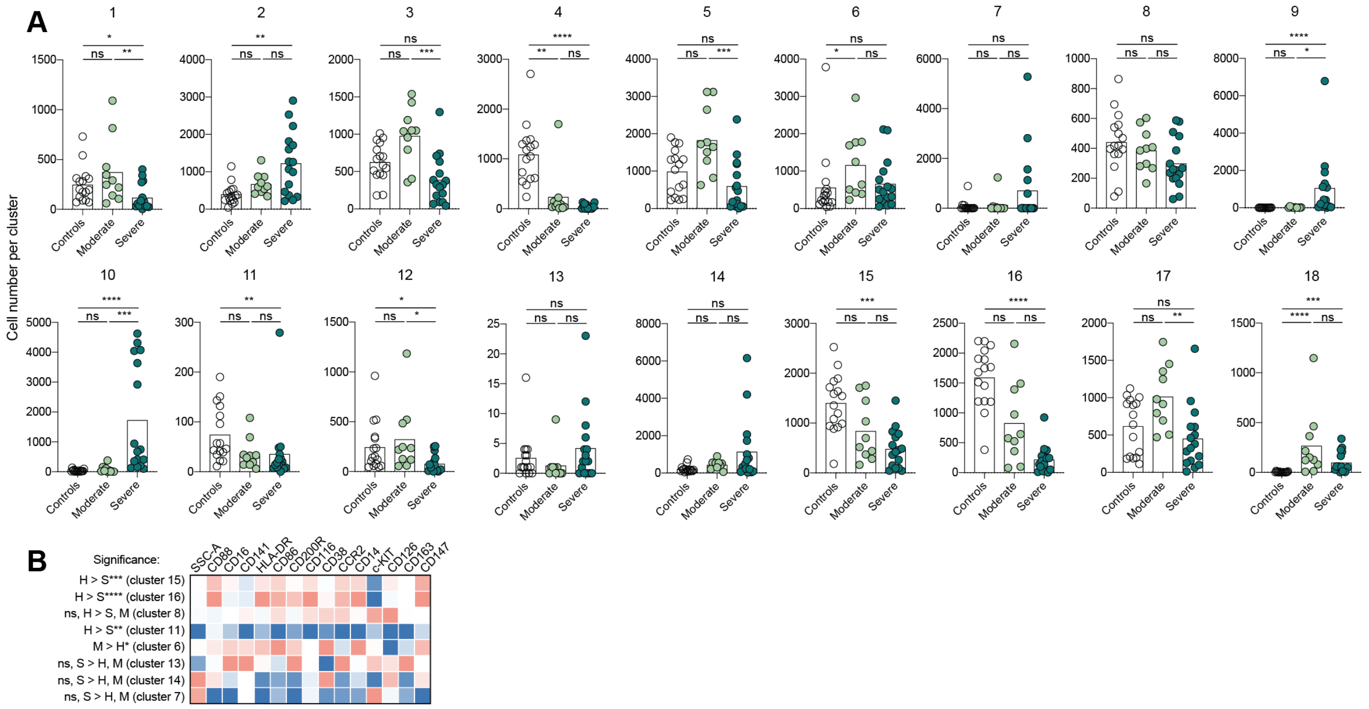

Fig. S2. Monocyte clusters (related to Fig. 4)

(A) Differences in cell numbers per Phenograph cluster 1-18 among the three cohorts. (B) Expression of markers in the remaining Phenograph clusters among the three cohorts. Statistical evaluation using Kruskal-Wallis test and Dunn's multiple comparisons test for comparison between the three cohorts. Significance level: *$p<0.05$, **$p<0.01$, ***$p<0.001$, ****$p<0.0001$

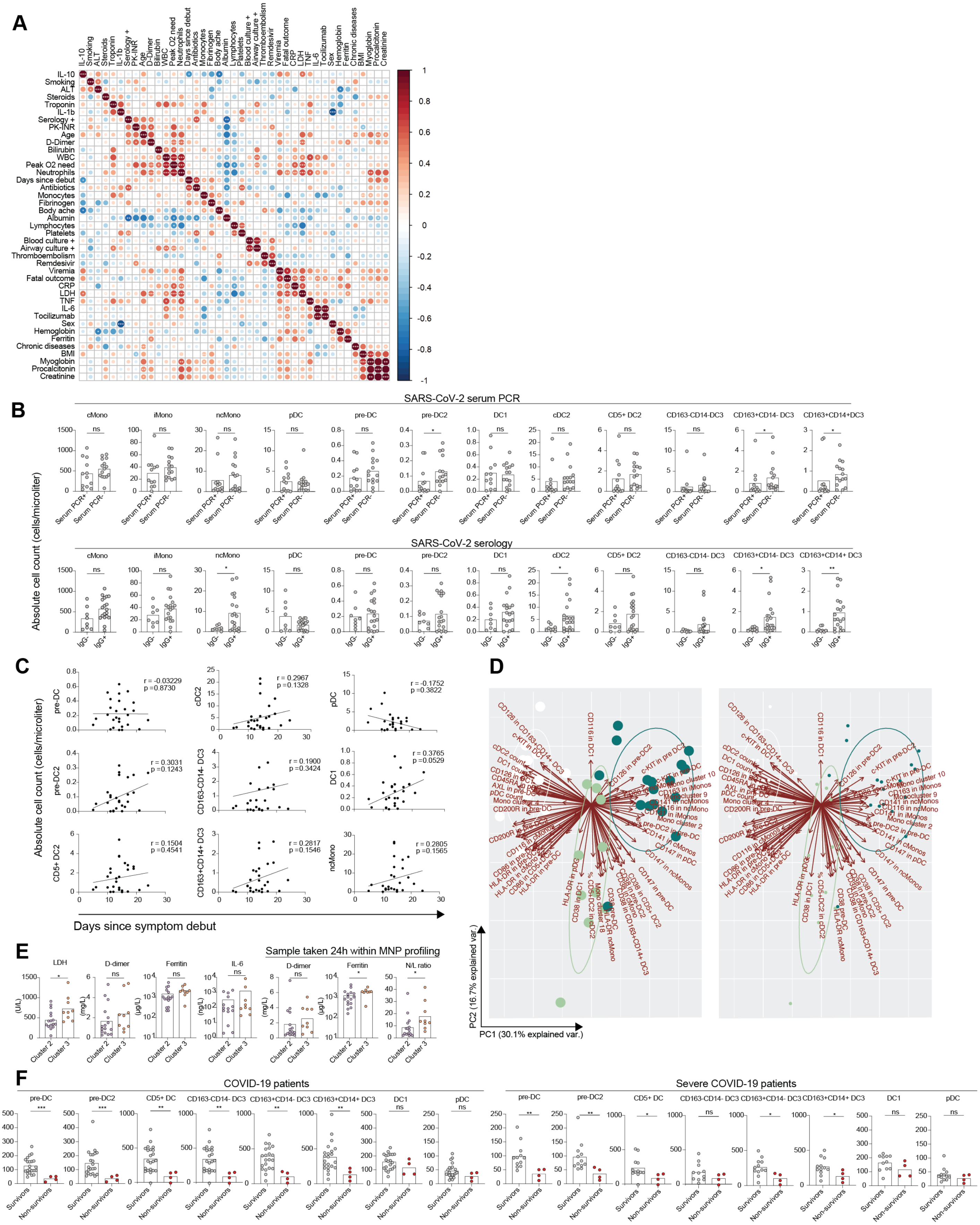

Fig S3. Clinical hyperinflammation, MNP landscape, and COVID-19 outcome (related to Fig. 5)

(A) Integrated correlation clustering of clinical parameters with CRP, procalcitonin, hemoglobin, WBC, lymphocytes, neutrophils, ferritin, troponin, D-dimer, PK-INR, fibrinogen, creatinine, bilirubin, and albumin taken within 24 hours from the MNP profiling; the color of the circles indicated positive (red) and negative (blue) correlations, color intensity represented correlation strength as measured by the Pearson's correlation coefficient. (B) Differences in absolute numbers of MNP populations between SARS-CoV-2 PCR+ and SARS-CoV-2 PCR- patients, and between SARS-CoV-2 IgG- and SARS-CoV-2 IgG+ patients. (C) Correlation of days since symptom debut until the sample collection and absolute numbers of cells within MNP populations. (D) Principal component analysis of 108 MNP parameters in the three cohorts, parameters contributing most to the separation highlighted; each dot represents controls (white), moderate COVID-19 (light green), and severe COVID-19 (dark green) patient; to better visualize marker names, a plot with smaller dots is provided (right). (E) Differences in LD, D-dimer, Ferritin, neutrophils/lymphocyte ratio at peak or 24h within MNP profiling (lower row) between patient cluster 2 and 3. (F) Differences in CD200R MFI between survivors and non-survivors in all patients (left) and in severe patients only (right). Statistical evaluation using Mann-Whitney for comparison between the two groups, Spearman test for correlations of non-normally distributed data and Pearson test for correlations of normally distributed data. Significance level: *$p<0.05$, **$p<0.01$, ***$p<0.001$, ****$p<0.0001$.

Table S1. Clinical characteristics

	Moderate	Severe
Group size, n	10	17
**Risk factors**		
Age, median (range)	56.5 (18-76)	58 (40-78)
Sex, n (female/male)	3/7	3/14
BMI, median (range)	27.63 (23- 35.06)	29 (23-55)
Smoking, n (%) -current	1 (10)	2 (12)
- prior	2 (20)	5 (29)
- non-smoker	2 (20)	9 (53)
- unknown	5 (50)	1 (6)
**Comorbidities, n (%)**		
None	4 (40)	5 (29)
Hypertension	1 (10)	6 (35)
Diabetes	3 (30)	5 (29)
Chronic heart disease	1 (10)	2 (12)
Asthma	1 (10)	2 (12)
Other	Obstructive sleep apnea syndrome (OSAS)	Renal failure, OSAS, Latent Tuberculosis, Chronic hepatitis B infection
**Hospitalization details**		
Days with symptoms prior to admission, mean (range)	8.7 (4-14)	8.6 (3-14)
Days with symptoms prior to sampling, mean (range)	12.9 (6-19)	14.6 (5-24)
Incidence of bacterial infection, n (%)	0 (0)	4 (24)
**Peak supportive oxygen therapy during hospitalization, n (%)**		
None	2 (20)	0 (0)
Low flow 0.5-10L/min	6 (60)	0 (0)
Low flow 10–15 L/min	1 (10)	3 (18)
High Flow	1 (10)	1 (6)
Ventilator	0 (0)	12 (71)
ECMO	0 (0)	1 (6)
**Lung function/severity scores at sampling**		
PaO_2/FiO_2 (PF) ratio mmHg, mean (range)	369 (312-523)	139 (52-285)
SOFA respiration*, median (range)	1 (0-1)	3 (2-4)
SOFA score, median (range)	1 (0-2)	6 (2-12)
NIH Ordinal Scale, median (interquartile range)	5 (5-5)	7 (6-7)
**Outcome**		
Discharged, n (%)	10 (100)	12 (71)
Deceased, n (%)	0 (0)	4 (24)
Ongoing treatment, n (%)	0 (0)	1# (6)

* Part in SOFA score regarding respiratory system.
\# Still in ECMO (19 August 2020).
BMI, body mass index; ECMO, extracorporeal membrane oxygenation; IL, interleukin; NA not available; TNF, tumor necrosis factor.

Table S2. Individual patient characteristics

Patient	Severity group	Days from symptom debut to admission	Days from symptom debut to sampling	Blood culture +/- 5 days from sampling	Culture finding in lower resp. secr. +/- 5 days from sampling	Steroids*	Antibiotics*	COVID-19 therapy*
Patient 1	Moderate	7	14	0	0	No	Cefotaxim	0
Patient 2	Moderate	7	14	0	0	Yes	Cefotaxim	0
Patient 3	Moderate	12	17	0	0	No	0	0
Patient 4	Moderate	8	10	0	0	No	0	0
Patient 5	Moderate	10	13	0	0	No	0	0
Patient 6	Moderate	7	14	0	0	No	0	0
Patient 7	Moderate	4	6	0	0	No	0	0
Patient 8	Moderate	9	11	0	0	No	0	0
Patient 9	Moderate	14	19	0	0	Yes	Cefotaxim	0
Patient 10	Moderate	9	11	0	0	No	0	0
Patient 11	Severe	7	14	0	0	No	Cefotaxim	0
Patient 12	Severe	7	14	0	S. dysgalactiae, S. aureus	No	0	0
Patient 13	Severe	9	12	S. aureus	S. aureus	Yes	0	0
Patient 14	Severe	3	11	S. milleri	S. aureus, K. pneumoniae, A. fumigatus, C. albicans	Yes	Cefotaxim	0
Patient 15	Severe	5	12	0	0	Yes	Piperacillin/Tazobactam	0
Patient 16	Severe	14	19	0	0	No	Cefotaxim	0
Patient 17	Severe	12	16	0	0	Yes	Cefotaxim	0
Patient 18	Severe	13	20	0	0	Yes	Cefotaxim	0
Patient 19	Severe	4	5	0	0	No	0	0
Patient 20	Severe	7	13	S. aureus	S. aureus, H. influenzae	No	0	Remdesivir
Patient 21	Severe	9	15	0	0	Yes	Cefotaxim	Tocilizumab
Patient 22	Severe	6	10	0	0	Yes	0	0
Patient 23	Severe	11	15	0	0	Yes	Cefotaxim	0
Patient 24	Severe	12	17	0	0	Yes	Cefotaxim	Anakinra
Patient 25	Severe	4	24	0	E. coli	Yes	Cefotaxim, Piperacillin/Tazobactam	0
Patient 26	Severe	14	17	0	C. albicans	Yes	Cefotaxim	0
Patient 27	Severe	9	14	S. aureus, E. faecalis	S. aureus	Yes	Cefotaxim	0

* Started at hospital prior to sampling

Table S3. Antibodies

Laser	Filter	Fluorophore	Antigen	Clone	Dilution	Company	Function or marker
	379/28	BUV395	CD123	7G3	100	BD	DC subsets
	515/30	BUV496	AXL	108724	25	BD	DC subsets
	580/20	BUV563	CD147	HIM6	50	BD	Metalloproteinase inducer, suspected spike protein
UV	605/20	BUV615	CCR2	LS132.1D9	100	BD	Chemokine receptor
(355nm)	670/25	BUV661	CD38	G46-6	200	BD	Ecto-enzyme
	735/30	BUV737	CD16	3G8	400	BD	Monocyte subsets
	810/40	BUV805	CD86	2A9-1	25	BD	Costimulatory molecule
	450/50	BV421	CD200R	OX-108	25	Biolegend	Inhibitory receptor
	525/50	BV510	CD15	W6D3	100	Biolegend	Neutrophil/eosinophil marker
	525/50	DCM Aqua	Dead cell stain	-	100	Thermo fisher	Dead cell marker
	586/15	BV570	CD44	IM7	200	Biolegend	CD147 binding partner
	605/40	BV605	FceR1	AER-37	100	BD	DC subsets
	677/20	BV650	CD163	GHI/61	50	BD	DC subsets
Violet	710/50	BV711	CD141	1A4	50	BD	Thrombomodulin/DC subsets
(405nm)	750/30	BV750	CD34	581	100	BD	Stem cell marker
	750/30	BV750	CD3	SK7	50	Biolegend	T cell marker
	750/30	BV750	CD7	M-T701	50	BD	T, NK, ILC marker
	750/30	BV750	CD19	HIB19	50	BD	B cell marker
	810/40	BV786	HLA-DR	L243	50	Biolegend	MHC class II receptor
Blue	530/30	BB515	CD206	19.2	50	BD	Mannose receptor
(488nm)	710/50	BB700	CD1c	F10/21A3	50	BD	Antigen-presenting protein
	586/15	PE	CD116	4H1	100	Biolegend	GM-CSF receptor
Yellow-	610/20	PE-CF594	CD126	M5	50	BD	IL-6 receptor
green	670/30	PE-Cy5	CD5	UCHT2	100	BD	DC2, pre-DC marker
(561nm)	710/50	PE-Cy5.5	c-KIT	95C3	25	Beckman Coulter	Stem cell growth factor receptor
	780/60	PE-Cy7	CD88	S5/1	200	Biolegend	Complement C5a receptor
	670/30	AF647	CLEC9A	3A4	50	BD	DC subsets
Red	730/45	AF700	CD45RA	HI100	100	BD	Activation marker
(637nm)	780/60	APC-Cy7	CD14	M5E2	100	Biolegend	Endotoxin receptor

Members of Karolinska KI/K COVID-19 Study Group

John Tyler Sandberg
Helena Bergsten
Niklas K Björkström
Susanna Brighenti
Marcus Buggert
Marta Butrym
Benedict J Chambers
Puran Chen
Martin Cornillet
Angelica Cuapio
Isabel Diaz Lozano
Majda Dzidic
Johanna Emgård
Malin Flodström-Tullberg
Jean-Baptiste Gorin
Sara Gredmark-Russ
Alvaro Haroun-Izquierdo
Laura Hertwig
Sadaf Kalsum
Jonas Klingström
Efthymia Kokkinou
Egle Kvedaraite
Hans-Gustaf Ljunggren
Nicole Marquardt
Magdalini Lourda
Kimia T Maleki
Karl-Johan Malmberg
Jakob Michaëlsson
Jenny Mjösberg
Kirsten Moll
Jagadeeswara Rao Muvva
Anna Norrby-Teglund
Laura M Palma Medina
Tiphaine Parrot
Lena Radler
Emma Ringqvist
Johan K Sandberg
Takuya Sekine
Tea Soini
Mattias Svensson
Janne Tynell
Andreas von Kries
David Wullimann
André Perez-Potti
Olga Rivera-Ballesteros
Christopher Maucourant
Renata Varnaite
Mira Akber

Lena Berglin
Demi Brownlie
Marco Giulio Loreti
Ebba Sohlberg
Tobias Kammann
Elisabeth Henriksson
Kristoffer Strålin
Soo Aleman
Anders Sönnerborg
Lena Dillner
Anna Färnert
Hedvig Glans
Pontus Nauclér
Olav Rooyackers
Johan Mårtensson
Lars I Eriksson
Björn P Persson
Jonathan Grip
Christian Unge
